# SPSNet: subpopulation-sensitive network-based analysis of heterogeneous gene expression data

**DOI:** 10.1186/s12918-018-0538-1

**Published:** 2018-03-19

**Authors:** Abha Belorkar, Rajanikanth Vadigepalli, Limsoon Wong

**Affiliations:** 1 School of Computing, National University of Singapore, 13 Computing Drive, Singapore, 117417 Singapore; 2 Daniel Baugh Institute for Functional Genomics and Computational Biology, Department of Pathology, Anatomy, and Cell Biology, Thomas Jefferson University, 1020 Locust Street, Philadelphia, 19107 Pennsylvania USA

**Keywords:** SPSNet, Heterogeneity, Gene expression, Differential expression analysis

## Abstract

**Background:**

Transcriptomic datasets often contain undeclared heterogeneity arising from biological variation such as diversity of disease subtypes, treatment subgroups, time-series gene expression, nested experimental conditions, as well as technical variation due to batch effects, platform differences in integrated meta-analyses, etc. However, current analysis approaches are primarily designed to handle comparisons between experimental conditions represented by homogeneous samples, thus precluding the discovery of underlying subphenotypes. Unsupervised methods for subtype identification are typically based on individual gene level analysis, which often result in irreproducible gene signatures for potential subtypes. Emerging methods to study heterogeneity have been largely developed in the context of single-cell datasets containing hundreds to thousands of samples, limiting their use to select contexts.

**Results:**

We present a novel analysis method, SPSNet, which identifies subtype-specific gene expression signatures based on the activity of subnetworks in biological pathways. SPSNet identifies the gene subnetworks capturing the diversity of underlying biological mechanisms, indicating potential sample subphenotypes. In the presence of extrinsic or non-biological heterogeneity (e.g. batch effects), SPSNet identifies subnetworks that are particularly affected by such variation, thus helping eliminate factors irrelevant to the biology of the phenotypes under study.

**Conclusion:**

Using multiple publicly available datasets, we illustrate that SPSNet is able to consistently uncover patterns within gene expression data that correspond to meaningful heterogeneity of various origins. We also demonstrate the performance of SPSNet as a sensitive and reliable tool for understanding the structure and nature of such heterogeneity.

**Electronic supplementary material:**

The online version of this article (10.1186/s12918-018-0538-1) contains supplementary material, which is available to authorized users.

## Background

Diseases and biological processes are highly heterogeneous due to variation in the underlying mechanisms. Regardless of its origin, heterogeneity is often implicit and undeclared, as incomplete knowledge prevents the accurate identification of subpopulations in a phenotype. Undeclared heterogeneity in transcriptomic data can arise from biological variation such as diversity of disease subtypes, treatment subgroups, time-series gene expression, nested experimental conditions, as well as technical variation due to batch effects, platform differences in integrated meta-analyses, etc. Unless the underlying heterogeneity is appropriately considered, comprehensive analysis of disease mechanisms is hindered, potentially resulting in misleading conclusions. In general, a systematic understanding of the biological basis of heterogeneity is critical in many practical contexts, e.g.:

- developing effective treatments by precise identification of dysregulated mechanisms in distinct disease subtypes.
- identifying differences in the molecular states of stem cells resulting in distinct lineage progression, to better understand organ development and regeneration; and
- detecting and eliminating the effects of intrinsic heterogeneity (e.g., cell cycle differences across cells, variation in cellular composition), which can hinder the discovery of physiologically relevant variation in the gene expression profiles.

A systematic analysis of non-biological and extrinsic heterogeneity is also useful in many cases, even when analyzing apparently homogeneous experimental conditions, for:

- extracting knowledge with greater confidence from a meta-analysis of independently generated datasets;
- discovering unsuspected anomalies or technical errors; and
- identifying and eliminating factors most influenced by extrinsic elements and/or batch effects.

Yet, handling heterogeneity in gene expression is a major problem with few and ineffective solutions. Previous studies have attempted to unravel heterogeneity using unsupervised techniques to identify gene expression-based, subtype-specific, molecular signatures [1–4]. In these approaches, gene expression data is typically subjected to hierarchical clustering or orthogonal transformation, and subpopulations in the sample are inferred using observations on the patterns of variation in gene expression. However, analysis carried out at the individual-gene level prevents a systemic view of the underlying mechanisms, and leaves considerable room for subjective, and potentially incorrect, interpretation of the underlying biological mechanisms. It also leads to a high false-positive rate, and low reproducibility [5]. Notably, Venet el al. showed that, in case of breast cancer, such gene-based signatures are no more reproducible than randomly chosen signatures [6].

Several methods have been proposed for analyzing differential expression between homogeneous phenotypes at the level of biological pathways and subnetworks, including Over-Representation Analysis (ORA) [7], Gene Set Enrichment Analysis (GSEA) [8], Gene Graph Enrichment Analysis (GGEA) [9], and Differential Expression Analysis in Pathways (DEAP) [10]. However, it has been demonstrated that, when analyzing independent datasets consisting of identical phenotypes, these methods produce results that considerably differ between the independent datasets, demonstrating lack of consistency. This issue arises mainly due to ineffective data normalization and/or the utilization of incorrect null hypothesis/distribution. Two recent methods overcome these issues to yield consistent results across data sets: SNet[11] and its refinement PFSNet[12]. However, these methods are designed to analyze only homogeneous phenotypes without subclasses.

We propose a generalized approach to analyze heterogeneity in gene expression data, and obtain subtype-specific signatures based on the differential gene expression of subnetworks in biological pathways rather than individual genes. Our generalization of PFSNet is termed SPSNet (SubPopulation-sensitive PFSNet). While PFSNet reports subnetworks that are differentially expressed between two samples representing homogeneous phenotypes, SPSNet makes no assumptions on the homogeneity of given phenotypes and automatically identifies subnetworks that are differentially expressed between the subpopulations within phenotypes. Thus, SPSNet serves a two-fold purpose: (i) when heterogeneity is biological in nature, it provides insights into how subpopulations within a sample set indicating diverse biological mechanisms manifest as sample subphenotypes; and (ii) in the presence of extrinsic or non-biological heterogeneity, our method amplifies these effects, facilitating identification and elimination of factors extraneous to biology of the phenotypes being studied. We demonstrate the utility and performance of our method using publicly available gene expression datasets containing disease heterogeneity, batch effects, and varied experimental treatments.

## Methods

### Data

- Leukemia dataset by Yeoh et al. [13]: We use the normal class (12 training, 6 test patients) and two large ALL subtypes, TEL-AML1 (52 training, 25 test patients), T-ALL (29 training, 15 test patients) from this microarray dataset.
- Hepatocellular Carcinoma (HCC) dataset by Roessler et al. [14]: This microarray dataset consists of 247 tumor and 241 adjacent non-tumor samples.
- HCC dataset by Burchard et al. [15]: This microarray dataset consists of 268 tumor and adjacent 249 non-tumor samples.
- TCGA RCC dataset—[16]: This microarray dataset contains 30 normal and 30 clear cell Renal Cell Carcinoma (ccRCC) tumor samples.
- Rat Toxicogenomics dataset by Wang et al. [17]: This RNA-Seq dataset contains 105 rat livers treated with 27 different chemicals representing 6 modes of action.
- We obtained human pathway information from the PathwayAPI database which consists of 300 human pathways [18] (available as Additional file 1 within the article’s additional material). The rat pathway information was obtained from the KEGG database [19] (available as Additional file 2).

### Notations and terminology

- *G*: the set of all genes *g*_*i*_ (*i*∈{1,2,…,*n*}) whose expression has been measured
- *P*_*C*_, *P*_¬*C*_: set of patients in the control and test phenotypes respectively, where the phenotypes potentially contain undeclared sources of heterogeneity. The objective of SPSNet is to identify gene subnetworks that are significantly differentially expressed between *P*_*C*_ and *P*_¬*C*_, while accounting for this potential heterogeneity.
- *E*(*g*,*p*): expression value of gene *g* in patient *p*
- *F*(*g*,*p*): the fuzzy score of gene *g* in patient *p*, as obtained by applying a GFS transform [20] on the gene expression matrix. Briefly, genes are ranked in each patient according to their raw expression, and a fuzzy score is obtained by using two thresholds *θ*_1_ and *θ*_2_; genes in the upper *θ*_1_ quantile are assigned a score of 1, genes below the *θ*_2_ quantile are assigned a score of 0, and those in between are assigned a score by linear interpolation. In our earlier work [20], we demonstrated that this transformation leads to great improvement in the quality of downstream analysis, as compared to preprocessing by mean-scaling, z-score, and quantile normalization.
- *β*(*g*,*X*): the relevance factor of gene *g* in a population represented by a set of patients *X*. The factor denotes how consistently *g* gets highly expressed in *X*, and is computed as the average fuzzy score of *g* over all patients in *X*:
  1 $$ \beta(g, X) = \sum\limits_{p \in X}{\frac{{F(g, p)}}{\vert X \vert}}  $$
- *S*: the set of all candidate subnetworks *S*_*k*_ (*k*∈{1,2,…,*r*}) generated from known biological pathways.

### Approach

#### Generating candidate subnetworks

The primary goal of SPSNet is to identify biological factors that distinguish subpopulations within a sample. Therefore, pathways were chosen to generate subnetworks as they represent the biological processes in an organism, and differences in their functioning contribute to differences within phenotypes. SPSNet does not preclude generating subnetworks from high-quality PPI networks. Both PPI networks and biological pathways can be supplied, even simultaneously, as input to SPSNet (and also to PFSNet). However, in the present manuscript, we do not investigate PPI networks since there are confounding issues when using PPI networks. For example, a PPI network is strictly speaking an artificial assembly of pairwise PPIs: While each individual PPI is a real biological interaction, the subnetwork itself is misleading because e.g. not all partners of a protein in the subnetwork actually simultaneously bind the protein. To ensure a straightforward interpretation and evaluation of our method, we prefer to exclude PPI networks in this manuscript.

The standard PFSNet methodology uses highly expressed genes from each phenotype to induce subnetworks on known biological pathways. However, this technique for generating candidate subnetworks is not suitable for heterogeneous data, as the presence of multiple subpopulations in a phenotype is likely to dilute high expression in any specific subtype. Therefore, we generate subnetworks as in NEA [21]; i.e. we form a subnetwork from each gene and its immediate neighbors in a biological pathway. We filter out subnetworks with less than 5 genes. We generate a total of 5654 such subnetworks from 300 human pathways in PathwayAPI [18].

#### Computing subnetwork scores

A GFS tranform is first applied to the gene expression matrix, as described in “Methods” section. All subnetworks are then assigned phenotype-wise scores for each patient as follows. A subnetwork *S*_*k*_ is scored in phenotype *C* by summing the fuzzy votes of all patients towards each member gene in *S*_*k*_, weighted by the respective gene relevance factors in *C*. Similarly, a score corresponding to ¬*C* is obtained by weighing the gene fuzzy votes with the respective relevance factors in ¬*C*. With the null hypothesis that subnetwork *S*_*k*_ is not relevant to difference between phenotypes *C* and ¬*C*, we test whether distribution of the difference between their corresponding scores is centered around zero. In particular,

2 $$\begin{array}{*{20}l} PScore(p, S_{k}, C) &= \sum\limits_{g \in S_{k}}{F(g, p) \times \beta(g, C)} \end{array} $$

3 $$\begin{array}{*{20}l} PScore(p, S_{k}, \neg C) &= \sum\limits_{g \in S_{k}}{F(g, p) \times \beta(g, \neg C)} \end{array} $$

Since PFSNet assumes no underlying heterogeneity in the phenotypes, the two relevance factors *β*(*g*,*C*) and *β*(*g*,¬*C*) are computed using the average of fuzzy votes in *all* patients in the respective phenotype. However, since SPSNet deals with heterogeneous data, we wish to compute subpopulation-specific relevance factors, rather than relevance factors over entire phenotypes. For this, we assume that each subpopulation in a phenotype has at least one subnetwork for which it has the highest expression among members of the phenotype. We then select *representative patients* for each subpopulation as the top *x* patients with highest expression of the subnetwork (supposing that the smallest subpopulation has at least *x* members), and use these to compute the subpopulation specific relevance factors. In our analysis, we set the value of *x* to 10, unless specified otherwise.

For each subnetwork *S*_*k*_, we compute the sum of gene fuzzy votes in patients belonging to both phenotypes *C* and ¬*C*. Thus, two vectors *V*(*S*_*k*_,*C*) and *V*(*S*_*k*_,¬*C*) are generated as:

4 $$ {}V(S_{k}, C) = \left[\sum_{g \in S_{k}}{F(g, p_{1})}, \sum_{g \in S_{k}}{F(g, p_{2})}, \dots, \sum_{g \in S_{k}}{F(g, p_{\vert C \vert})}\right]  $$

5 $$ {}V(S_{k}, \neg C)\! \,=\,\! \left[\!\sum_{g \in S_{k}}{\!F\!\left(g, p'_{1}\right)},\! \sum_{g \in S_{k}}{\!F\!\left(g, p'_{2}\right)}, \dots,\!\! \sum_{g \in S_{k}}{\!F\left(g, p'_{\vert \neg C \vert}\right)}\right]  $$

The top *x* patients each with the highest values in *V*(*S*_*k*_,*C*) and *V*(*S*_*k*_,¬*C*) are then selected as the *representative patients*. Let the set of these patients be denoted as *Q*(*S*_*k*_,*C*) and *Q*(*S*_*k*_,¬*C*) respectively. Then, we compute the final scores for each subnetwork as:

6 $$ {}SScore(p, S_{k}, C) = \sum\limits_{g \in S_{k}}{F(g, p) \times \beta(g, Q(S_{k}, C))}  $$

7 $$ {}SScore(p, S_{k}, \neg C) = \sum\limits_{g \in S_{k}}{F(g, p) \times \beta(g, Q(S_{k}, \neg C))}  $$

8 $$ {}{SPS \_ Score}(p, \!S_{k}, \!C, \!\neg C) \!\!= \!SScore(p, \!S_{k}, \!\neg C) - SScore(p, \!S_{k}, \!C)  $$

Similar to PFSNet, the null hypothesis in SPSNet is that subnetwork *S*_*k*_ is not relevant to difference between phenotypes *C* and ¬*C*. Therefore, it is tested whether the distribution of SPS_Score (as in Eq. (8)) is centered around zero. However, before testing the subnetworks for statistical significance, we eliminate candidate subnetworks which do not contain at least five genes with a phenotype-specific (subpopulation-specific) relevance factor greater than or equal to 0.5 in PFSNet (SPSNet). Setting this cutoff ensures that genes in each candidate subnetwork are highly expressed in at least half of the patients of that phenotype/subpopulation, and thus helps to reduce false positives.

#### Determining statistical significance

In the standard PFSNet methodology, a null score distribution for each phenotype is generated by randomly swapping class-labels between patients in the control and test samples, and computing subnetwork scores using the permuted labels. However, we use the theoretical t-distribution as our null distribution, as a class-label permutation approach is not practical for SPSNet. This is because the number of representative patients (recall *x* = 10) is insufficient for generating the necessary number of class-label permutations. We test how distant the mean score of each subnetwork is from zero (on either side), and thereby estimate the corresponding statistical significance. All subnetworks with *p*-value below a given threshold are reported as significant. In here, we use the customary significance threshold of 0.05.

#### SPSNet as the generalization of PFSNet

As stated earlier in “Data” section, SPSNet is a generalization of PFSNet. When a ‘subpopulation’ expands to accommodate the entire phenotype, and all patients in the phenotype can be considered *representative* of it, SPSNet is equivalent to PFSNet:

$$ \begin{aligned} &{}{SPS\_Score}(p, S_{k}, C, \neg C) \\ &{}= \sum\limits_{g \in S_{k}}{\!F(g, p) \!\!\times\! \beta(g, Q(S_{k}, \neg C))} \,-\,\!\! \sum\limits_{g \in S_{k}}{\!F(g, p)\!\!\times\!\beta(g, \!Q(S_{k}, \!C))}  \\ &{}= {PFS\_Score}(p, S_{k}, Q(S_{k}, C), Q(S_{k}, \neg C)) \end{aligned}  $$

**Fig. 1:**
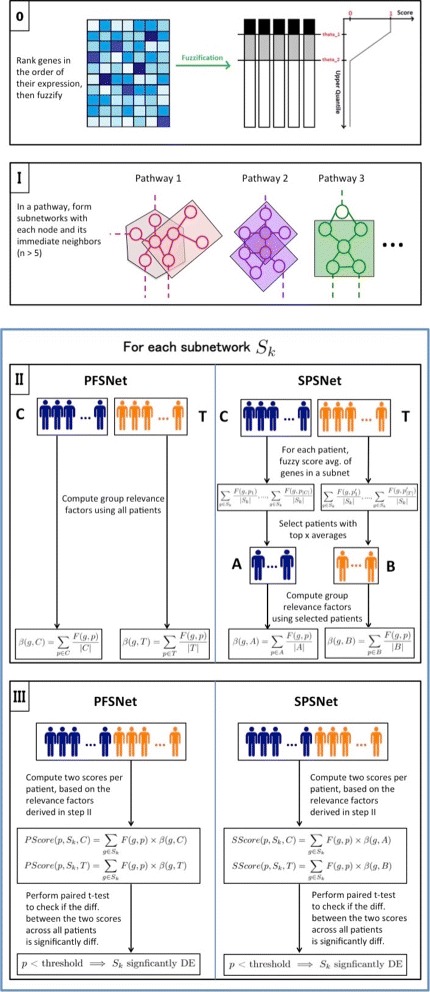
Flowchart illustrating the SPSNet methodology (in comparison to PFSNet)

An overview of the PFSNet and SPSNet methodology is presented in Fig. 1.

## Results and discussion

In analyzing the performance of SPSNet, we take a four-fold approach: (i) First, we merge samples with known experimental conditions; and test whether SPSNet is able to discover subnetworks known to be differentially expressed in the individual subpopulations in the merged dataset. We also quantitatively assess the discriminatory power of SPSNet by transforming the subnetwork scores into feature matrices, and computing silhouette scores on their PCA transform. (ii) To analyze the sensitivity and specificity of the method, we simulate test datasets with induced heterogeneity, and evaluate if SPSNet correctly identifies the differentially expressed subnetworks as such. (iii) To validate the reliability of SPSNet, we examine the overlap between subnetworks reported significantly differentially expressed on independent datasets with the same phenotype composition. (iv) Finally, we investigate whether the performance of SPSNet scales to datasets with greater heterogeneity using a dataset containing a variety of treatment groups.

### Comparison using homogeneous phenotypes

Since PFSNet performs well on homogeneous phenotypes [12], it is reasonable to assume that subnetworks reported by it when comparing two homogeneous classes are truly differentially expressed. Therefore, we compare the subnetworks reported significant from PFSNet runs on homogeneous classes, with those reported by SPSNet and PFSNet on heterogeneous classes obtained by merging multiple homogeneous phenotypes.

#### Acute Lymphoblastic Leukemia

**Fig. 2:**
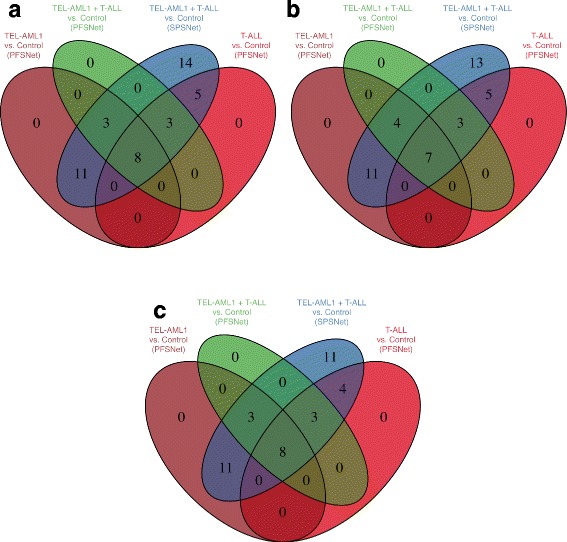
Acute Lymphoblastic Leukemia (ALL) – pathways containing differentially expressed subnetworks. **a** 30 TEL-AML1 + 29 T-ALL, **b** 30 TEL-AML1 + 20 T-ALL, **c** 30 TEL-AML1 + 10 T-ALL

We obtain subnetworks highly expressed in the TEL-AML1 subtype and are reported by PFSNet as significantly differentially expressed with respect to the normal class, and a similar set of subnetworks highly expressed in the T-ALL subtype. To simulate the heterogeneous case, we combine patients from both disease subtypes into a single “heterogeneous” disease class, and then obtain subnetworks highly expressed in it that are reported by PFSNet and SPSNet as significantly differentially expressed with respect to the normal class. Finally, we perform a pathway-level comparison of the subnetworks reported significant in the homogeneous and heterogeneous cases. Figure 2 records three sets of observations corresponding to datasets of increasing heterogeneity (where the disease sample is created by incrementally merging 10, 20, and 29 patients of the T-ALL subtype respectively, with 30 TEL-AML1 patients in each case). From the figure, we observe that both PFSNet and SPSNet are successful in identifying pathways common to the TEL-AML1 and T-ALL subtypes. However, SPSNet is more sensitive in detecting pathways that are specific to either of the disease subtypes.

#### Hepatocellular carcinoma

**Fig. 3:**
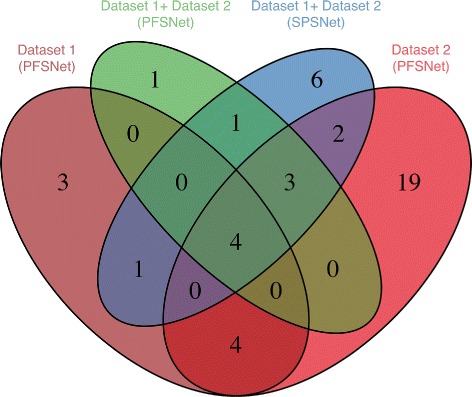
Hepatocellular Carcinoma (HCC) – pathways containing differentially expressed subnetworks that are highly expressed in HCC

We conduct a similar experiment on the two batches of HCC data, whereby subnetworks highly expressed in HCC and differentially expressed with respect to the normal sample are obtained for each batch separately, and after merging the two batches. Pathway-level comparison of these subnetworks is recorded in Fig. 3. We observe that PFSNet and SPSNet are able to discover pathways that have subnetworks differentially expressed in both HCC batches. However, SPSNet is able to better identify pathways differentially expressed only in one of the two batches, indicating its sensitivity to heterogeneity in samples.

### Estimating sensitivity and specificity from simulation

Simulation experiments, when carefully designed, have the advantage that ‘correct’ outcomes from the application of a method can be known in advance. Thus, they can be powerful tools for objective performance evaluation.

We simulate test samples with injected heterogeneity, pair them with homogeneous control samples, and compare subnetworks that are known to be differentially expressed between the two sample groups with those reported significant by SPSNet to estimate the sensitivity and specificity of our method. The detailed procedure is described below:

We choose a homogeneous normal sample, which is unlikely to contain any significantly differentially expressed genes at the outset. The normal sample is randomly split into two equal halves, *N*_1_ and *N*_2_, and one of these parts (*N*_2_) is allocated for injecting differential expression. To induce heterogeneity, *N*_2_ is further divided into two subtypes, *N*_21_ and *N*_22_, with *α*% and (100−*α*)% of its patients respectively. We sub-sample 10% of the total number of genes and induce differential expression in patients in *N*_21_ for these selected genes, in a manner similar to the description from Langley et al. [22]. i.e. we multiply the expression of patients in *N*_21_ by a factor of *r*, where *r* is chosen randomly from the set {1.2,1.5,1.8,2.0,3.0}, for each gene in the sub-sample. Another independent sub-sample of 10% genes is chosen, and differential expression corresponding to genes in this sub-sample is induced in patients belonging to the set *N*_22_.

Thus, we obtain four sets of genes, which we use to generate four sets of subnetworks:

- *G*_1_: genes differentially expressed between *N*_1_ and *N*_21_
- *G*_2_: genes differentially expressed between *N*_1_ and *N*_22_
- *G*_12_: genes differentially expressed between *N*_1_ and *N*_21_, AND between *N*_1_ and *N*_22_
- *G*_0_: genes not differentially expressed between *N*_1_ and *N*_21_ and between *N*_1_ and *N*_22_

To generate subnetworks from these genes, we adopt the procedure used by Goh et al. [23], emulating the feature of real biological subnetworks that genes in a subnetwork tend to have correlated expression patterns. In particular, we perform a hierarchical clustering of genes in *G*_1_, and reposition them within their clusters such that the most similar genes are next to each other. Subnetworks are then generated by splitting the resulting ordered list into sets of 7 genes each. A similar ordering after hierarchical clustering is obtained separately for *G*_2_,*G*_12_,*G*_0_. However, for *G*_0_, we do not use all the non-differentially expressed genes to form subnetworks, but only four times the number of genes in *G*_1_. This emulates the effect of incompleteness in biological pathway databases, and also saves computation time required to generate a vast number of negative control subnetworks.

The entire simulation process is repeated for 100 iterations. In each iteration, PFSNet and SPSNet are run on newly simulated data, and subnetworks generated from *G*_1_,*G*_2_,*G*_12_,*G*_0_ in the corresponding iteration are tested for significance.

#### Estimating sensitivity

We use two datasets for simulation, normal kidney and normal liver tissue expression data from TCGA [16] (Dataset 1) and Roessler et al. [14] (Dataset 2), which profile 20,502 and 13,801 genes respectively. The number of subnetworks generated in each iteration from Dataset 1 using *G*_1_,*G*_2_,*G*_12_,*G*_0_ are 292, 292, 30, 1168 respectively; while 197, 197, 20, 788 subnetworks are generated from Dataset 2. To understand the effect of different levels of heterogeneity within the data on the performance of PFSNet and SPSNet, we vary the parameter *α* in our simulations. For Dataset 1, *α* is set to 50% (the test sample is divided into two subtypes with 50% of its patients each), while for the larger Dataset 2, separate simulations are performed with *α* set to 20% (subtype 1 – 20%, subtype 2 – 80%), 40% (subtype 1 – 40%, subtype 2 – 60%), and 50% (subtype 1 – 50%, subtype 2 – 50%).

**Fig. 4:**
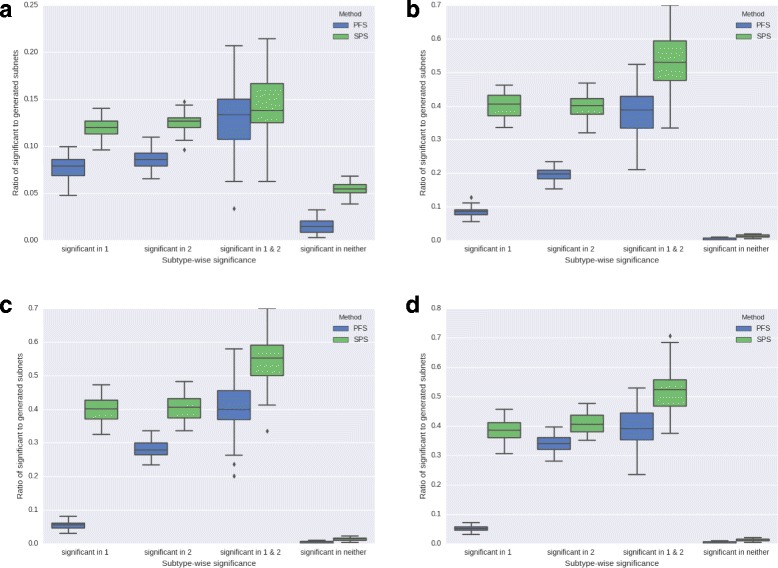
Proportion of significant subnetworks reported by PFSNet and SPSNet on test samples injected with different levels of heterogeneity. **a** Dataset 1: 50% subtype 1, 50% subtype 2, **b** Dataset 2: 50% subtype 1, 50% subtype 2, **c** Dataset 2: 40% subtype 1, 60% subtype 2, **d** Dataset 2: 20% subtype 1, 80% subtype 2

Figure 4a shows four boxplots for Dataset 1 corresponding to the fraction of subnetworks reported significant by PFSNet and SPSNet from subnetworks that are simulated to be significant in subtype 1, significant in subtype 2, simulated to be significant in both, and non-significant in both subtypes. Figure 4b to d show similar boxplots for Dataset 2, with varying levels of heterogeneity (different values of *α*).

As expected, both PFSNet and SPSNet show higher sensitivity for subnetworks significant in both subtypes, when compared with those significant in only one of the subtypes. In all three subnetwork categories—significant in subtype 1, subtype 2, and both—the sensitivity of SPSNet is higher than PFSNet (SPSNet improves the median sensitivity by about 10% in each case). The subnetworks not significant in either subtypes are rarely reported significant by PFSNet and SPSNet (high specificity); the false-positive rate, although a little higher in SPSNet than PFSNet, is within or around the 5% bound in all cases.

It is also interesting to note the impact of varying heterogeneity on the sensitivity of the two methods for simulations on Dataset 2. We notice that the output of PFSNet is strongly dominated by the majority subtype, while SPSNet is relatively insensitive to the level of heterogeneity. Thus, when *α* is set to 50%, the median sensitivity of PFSNet for subnetworks significant in subtype 1 and 2 is about 10% and 20% respectively. When *α* is decreased to 40%, the median sensitivity for subnetworks significant in subtype 1 (minority) drops to below 5% and median sensitivity for subnetworks significant in subtype 2 (majority) rises to about 25%. At an even lower *α* of 20%, the recall for subnetworks significant in subtype 1 remains almost the same, while the median sensitivity for subtype 2 rises to about 35%. On the other hand, SPSNet performs relatively better at all levels of heterogeneity; irrespective of the value of *α*, it consistently shows a median sensitivity of about 40%.

#### Estimating false-positive rate

To assess whether the false-positive rate in SPSNet is well-controlled, we use the same simulation setup as that in the previous subsection. We generate 1000 subnetworks using *G*_0_. Since the genes in *G*_0_ are differentially expressed between neither *N*_1_ and *N*_21_, nor *N*_1_ and *N*_22_, no subnetworks generated from *G*_0_ are expected to be differentially expressed. We run SPSNet and test whether the subnetworks are reported to be differentially expressed. For this experiment, we used the normal tissues from one of the HCC datasets [14]. Since the sample is considered homogeneous, any subnetworks reported differentially expressed are considered false positives. To observe whether sample size affects false-positive rate, we randomly selected subsamples of size 240, 210, 180, 150, 120, 90, 60, and 30, fifty times each.

**Fig. 5:**
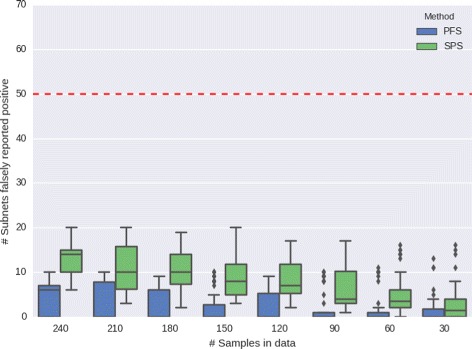
False-positive rate with varying sample size

Figure 5 shows boxplots depicting the range of false-positive rates corresponding to subsamples of each size. In samples of all sizes, the false positives were seen to be well-controlled: less than 50 of 1000 subnetworks are reported significant (FP rate < 0.05).

### Quality of feature selection

A good method for network-based differential expression analysis of heterogeneous data would report significant subnetworks that can serve as relevant features in distinguishing the classes being compared, as well as their component subpopulations. Therefore, we use the scores of significant subnetworks in PFSNet and SPSNet as features, and visualize scatter plots based on PCA transformation of the resulting feature matrices. Further, we quantitatively assess the ability of these features to distinguish between subpopulations, with silhouette scores computed using the feature matrices and known labels corresponding to patient subtype and/or subpopulation.

#### Acute Lymphoblastic Leukemia

**Fig. 6:**
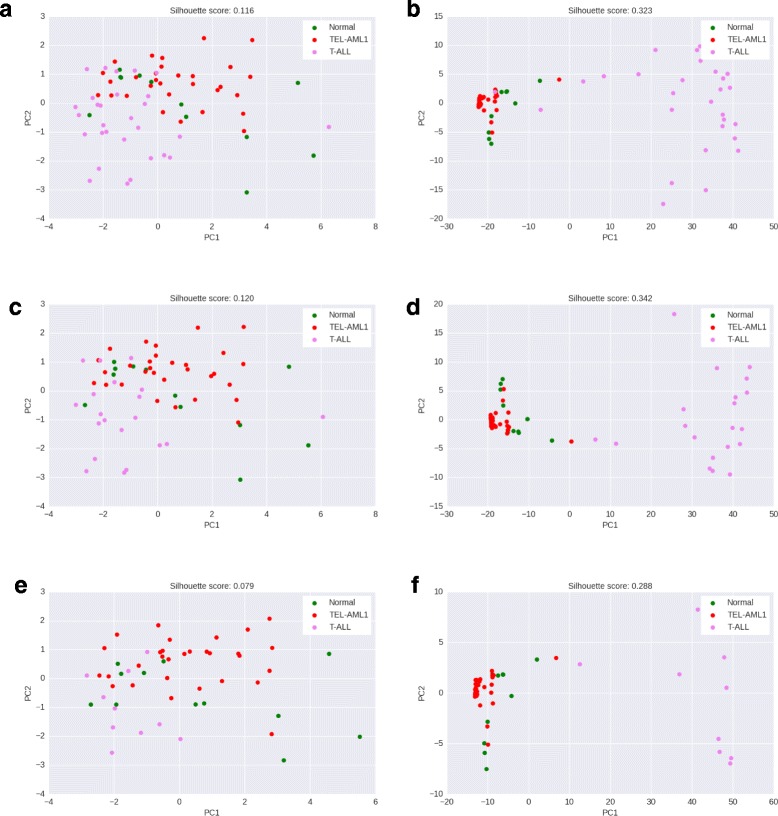
Normal vs heterogeneous ALL disease sample – PCA scatter plots based on scores of significant subnetworks in PFSNet and SPSnet. **a** PFSNet – (Normal vs. 30 TEL-AML1 + 29 T-ALL), **b** SPSNet – (Normal vs. 30 TEL-AML1 + 29 T-ALL), **c** PFSNet – (Normal vs. 30 TEL-AML1 + 20 T-ALL), **d** SPSNet – (Normal vs. 30 TEL-AML1 + 20 T-ALL), **e** PFSNet – (Normal vs. 30 TEL-AML1 + 10 T-ALL), **f** SPSNet – (Normal vs. 30 TEL-AML1 + 10 T-ALL)

We use the same samples as mentioned in previous sections with experiments on the ALL dataset [13] – normal class against datasets of increasing heterogeneity (where the disease sample is created by incrementally merging 10, 20, and 29 patients of the T-ALL subtype respectively, with 30 TEL-AML1 patients in each case). We draw PCA scatter plots corresponding to subnetworks reported as differentially expressed between normal and each heterogeneous disease sample (Fig. 6). Table 1 shows three sets of silhouette scores corresponding to feature matrices obtained from scores of significantly differentially expressed subnetworks reported on comparing normal sample with disease samples of increasing heterogeneity. From the silhouette scores, as well as PCA scatter plots of subnetwork scores, we observe that SPSNet is able to better discriminate between different disease subtypes within the ALL sample, across varying levels of heterogeneity.

**Table 1:** ALL – Silhouette scores based on the first 3 PCs of feature matrices built using scores significant subnetworks in PFSNet and SPSNet

	30 TEL-AML1	30 TEL-AML1	30 TEL-AML1
	+ 29 T-ALL	+ 20 T-ALL	+ 10 T-ALL
PFSNet	0.116	0.12	0.079
SPSNet	**0.323**	**0.342**	**0.288**

SPSNet leads to better separation (silhouette scores marked in bold) amongst subtypes in the ALL disease phenotype

#### Hepatocellular carcinoma

**Fig. 7:**
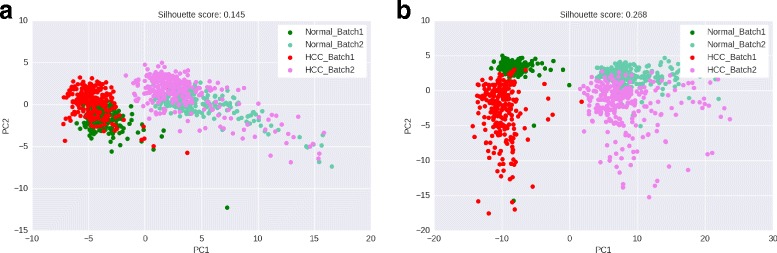
Normal vs HCC samples merged from two batches - PCA scatter plots based on scores of significant subnetworks in PFSNet and SPSnet. **a** PFSNet – PC1 and PC2, **b** SPSNet – PC1 and PC2

We use the two HCC datasets from [14] and [15], and create a new normal and HCC sample by merging the normal and disease samples respectively from both batches. PCA scatter plots drawn using scores of significant subnetworks are shown in Fig. 7a and c.

We observe that in the scatter plot corresponding to SPSNet features, patients appear better separated with respect to their batch as well as phenotype labels. Further, PC1 is able to capture and isolate almost all of the batch effects in the SPSNet scatter plot, whereas the batch effects spill over to the lower PCs in the case of PFSNet. This is despite the fact that PC1 in SPSNet covers only 66% of the total variance while PC1 in PFSNet covers 72% of its total variance. Thus, SPSNet proves to be effective at identifying the heterogeneity induced by batch effects.

Next, we eliminate PC1 to see if the normal and HCC samples (combined from two batches) can be clearly separated by the remaining PCs based on their phenotypes alone. From the silhouette scores in Table 2, it is seen that PC2 and PC3 from SPSNet features are able to better distinguish between normal and HCC samples, as compared to their counterparts from PFSNet features. These observations are in line with the remarks from our previous work [20] that eliminating PC1 often leads to removal of batch effects and a clearer separation based on phenotypes.

**Table 2:** HCC – Silhouette scores based on PCA transform applied to scores of subnetworks reported as significantly differentially expressed by PFSNet and SPSNet

	Normal vs HCC	Normal vs HCC
	(first 3 PCs, with batch labels)	(2^*nd*^, 3^*rd*^ PC, without batch labels)
PFSNet	0.145	0.117
SPSNet	**0.268**	**0.298**

SPSNet leads to better separation (silhouette scores marked in bold) between normal/HCC phenotypes, as well as the two different batches

### Reproducibility on independent datasets

A reliable method would produce significant subnetworks that agree highly when run on independent datasets with the same phenotypical composition. Therefore, we run PFSNet and SPSNet to obtain significantly differentially expressed subnetworks between normal sample and the heterogeneous ALL sample (with all patients from subtypes TEL-AML1 and T-ALL combined). This is done separately for the training and test data, and the agreement (in the form of jaccard coefficient) between significant subnetworks obtained on the two sets of data is recorded in Table 3. We observe that SPSNet shows much higher reproducibility on the heterogeneous dataset, as compared to PFSNet.

**Table 3:** Jaccard coefficients showing agreement between significant subnetworks obtained by PFSNet and SPSNet on training and test data

	Training	Test	Training ∩ test	Training ∪ test	Jaccard coefficient
PFSNet	27	24	11	40	**0.28**
SPSNet	87	77	62	102	**0.61**

Subnetworks reported significantly differentially expressed by SPSNet in the heterogeneous ALL phenotype are more reproducible (jaccard coefficients marked in bold) than PFSNet across training and test data

### Are *representative patients* of significant subnetworks enriched in specific subpopulations?

Since SPSNet utilises a subset of patients for each subnetwork to represent potential subpopulations in the phenotype, we study a) whether such subsets are enriched in one of the constituent subpopulations, and b) how such enrichment is affected by the relative proportions of the constituent subpopulations in the data.

**Fig. 8:**
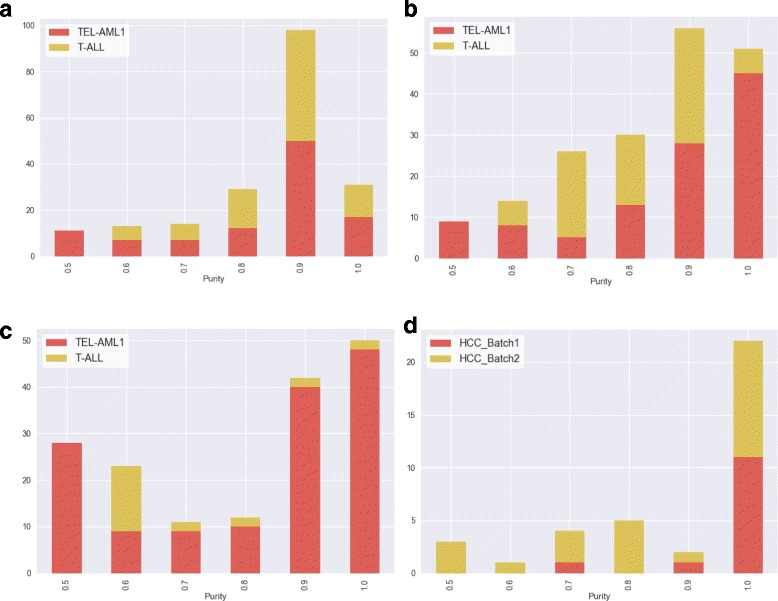
Number of subnetworks reported significant by SPSNet corresponding to different purity levels. A chi-squared test is performed to see if the number of significant subnetworks with high purity (purity > 0.75) is larger than those with low purity (purity ≤ 0.75); *p*-values are reported in brackets. **a** 30 TEL-AML1 + 29 T-ALL (p-val:1.1×10^−3^), **b** 30 TEL-AML1 + 20 T-ALL (p-val: 1.1×10^−10^), **c** 30 TEL-AML1 + 10 T-ALL (p-val: 1.02×10^−17^), **d** HCC merged dataset (p-val: 5.5×10^−4^)

To assess this, we once again use the ALL [13] and HCC datasets [14, 15], and define a measure ‘purity’ as the proportion of patients belonging to the majority subpopulation (subtype/batch) in the *representative patients* subset for a given significant subnetwork. Figure 8 records the number of significant subnetworks with purity levels between 0.5 to 1.0 and the colors indicate the majority subpopulation which resulted in the purity value.

We observe that a large proportion of significant subnetworks are enriched in one of the constituent subpopulations (high purity); such subnetworks help distinguish the subpopulations from each other. There are also a few significant subnetworks which have low purity (almost equal proportion of subpopulations); these indicate common biological characteristics shared by the subpopulations. Also, in the ALL dataset, when SPSNet is used to compare control sample with a heterogeneous disease sample containing 30 TEL-AML1 patients and 29 T-ALL patients, the contribution of the two disease subtypes to high purity levels (purity > 0.75) is similar; i.e. the number of significant subnetworks with representative patients having TEL-AML1 and T-ALL patients in majority is similar. This phenomenon persists even when the number of T-ALL patients is reduced to 20. However, when only 10 T-ALL patients are included in the heterogeneous sample, there are very few significant subnetworks with representative patients having a T-ALL majority. This suggests that SPSNet is able to recover minority subpopulations unless the size of the smaller subpopulations drops below a certain threshold (viz. *x*).

### Analysis on a dataset with more than two subgroups

The rat toxicogenomics RNA-Seq dataset [17], described in “Methods” section (Data), is an ideal test-bed for assessing the performance of SPSNet since the heterogeneity is experimentally-induced by treatment with different drugs, with strict control on potentially confounding variables such as drug concentration and medium of delivery. We extract two subsets from this dataset for our analysis: (A) a control group and a heterogeneous drug-treated group – 5 drugs with PPARA mode of action (Clofibric acid, Nafenopin, Bezafibrate, Rosiglitazone, Gemfibrozil), and (B) a control group and a heterogeneous drug-treated group – 6 drugs, each with a different mode of action (Clotrimazole, Ethinylestradiol, Simvastin, Chloroform, Leflunomide, Nafenopin). The dataset contains expression of three rat livers treated by each drug. We therefore set *x*, which is the number of representative patients selected per subnetwork, to a smaller value than the default (*x*=5).

**Fig. 9:**
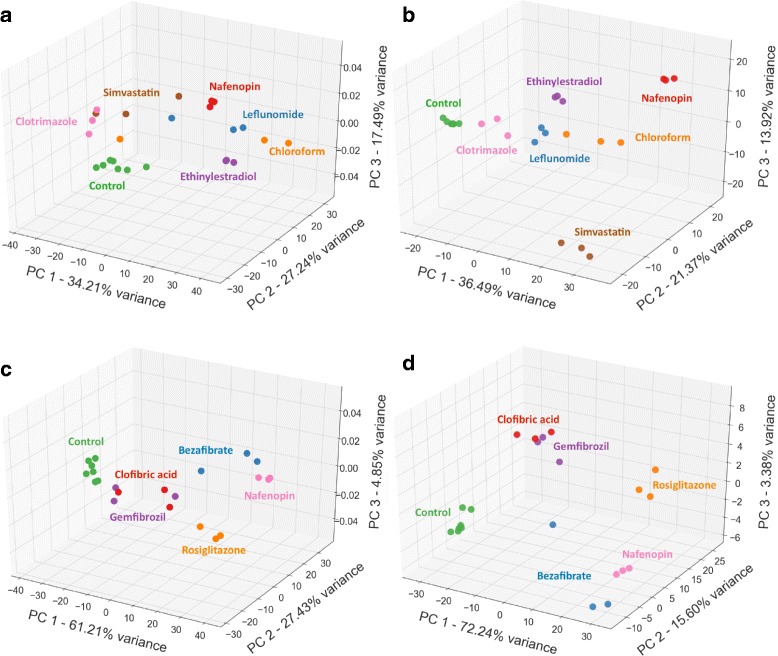
PCA transformation applied to SPSNet (subnetwork score) and ANOVA (gene expression) features captures the heterogeneity in drug treatment of rat livers. (Dataset of [17]). **a** SPSNet: across 6 modes of action, **b** ANOVA: across 6 modes of action, **c** SPSNet: Drugs representing PPARA mode of action, **d** ANOVA: Drugs representing PPARA mode of action

**Fig. 10:**
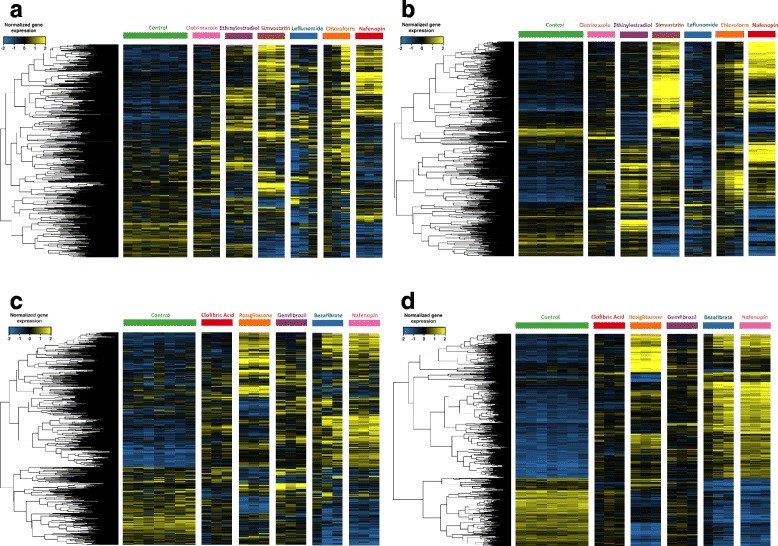
Heatmap showing similar expression patterns of significant genes (ANOVA), and genes in significant subnetworks (SPSNet), of rat livers treated with multiple drugs (Dataset of [17]). **a** SPSNet: across 6 modes of action, **b** ANOVA: across 6 modes of action, **c** SPSNet: Drugs representing PPARA mode of action, **d** ANOVA: Drugs representing PPARA mode of action

We ran SPSNet comparing the heterogeneous drug-treated groups to the control group. A PCA transform was applied to the scores of the subnetworks reported significant by SPSNet (Fig. 9a, c). Subnetworks with scores having a non-zero contribution to the first three principle components were selected and the genes contained in them were used to create the heatmaps in Fig. 10a, c.

Since the treatment labels are known apriori, we apply ANOVA to identify genes that distinguish between at least two treatment sub-groups. A significance threshold of FDR-corrected *p*-value of 10^−5^ was chosen to obtain a similar number of genes as those used to generate the heatmap in Fig. 10a, c (derived from SPSNet analysis). We then apply a PCA transform to the expression matrix of genes reported significant by ANOVA (Fig. 9b, d). These are further filtered to contain only those genes with non-zero contribution to the first three principle components. Heatmaps generated by the genes are shown in Fig. 10b, d.

Note that the labels of constituent subpopulations in the heterogeneous drug treated group are provided as an input to ANOVA, while SPSNet is not supplied with this information. Still, the PCA scatter plot of SPSNet subnetwork features show remarkable separation between different drug samples. Also, the heatmap patterns of genes obtained from SPSNet analysis show a resemblance to those of the ANOVA heatmaps.

Interestingly, the relative placement of treatment subgroups of rat livers remain consistent in the PCA scatter plots of ANOVA and SPSNet. In case of drugs across 6 different modes of action (Fig. 9a, b), we see that: (i) the drug action of Clotrimazole is mild, and its expression pattern shows close resemblance to control rat liver group; (ii) the drugs Ethinylestradiol, Leflunomide, and Chloroform induce similar responses in the liver.

The consistency continues to hold even in the heterogeneity analysis of drugs within the PPARA mode of action (Fig. 9c, d) – for analysis on both ANOVA and SPSNet features, we observe that (i) the actions of Clofibric acid and Gemfibrozil drugs are indistinguishably similar to each other; (ii) Nafenopin and Bezafibrate induce similar liver response but are marked by minor differences; (iii) the action of Rosiglitazone is remarkably different from the other PPARA drugs under comparison.

### Effect of varying number of representative patients on the performance of SPSNet

For each subnetwork, representative patients are chosen by SPSNet to ensure representation of a potential subpopulation in which the subnetwork is highly expressed. Ideally, the number of representative patients, say *x*, would be lower than or equal to the number of patients in the smallest subpopulation within the phenotype. Thus, when top *x* patients with the highest expression of a given subnetwork are chosen, the selected patients would likely belong to the same subpopulation.

**Fig. 11:**
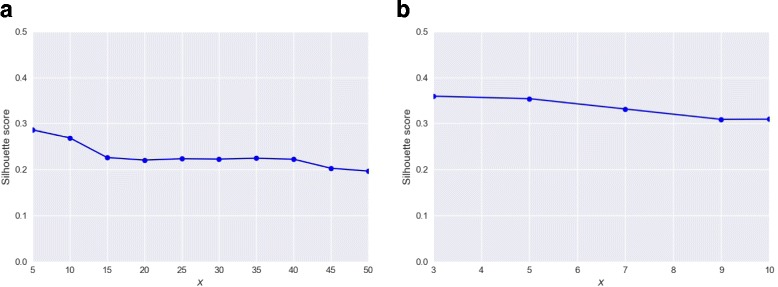
Effect of varying x (number of representative patients) in SPSNet on silhouette scores. **a** HCC merged dataset, **b** Drugs representing PPARA mode of action

Figure 11a and b show effect of varying the parameter *x* on the performance of SPSNet, in terms of its ability to distinguish between subpopulations based on subnetworks reported to be differentially expressed. A PCA transform was applied to the SPSNet scores of differentially expressed subnetworks, and a silhouette score was computed based on the first three principal components.

## Conclusion

Presence of undeclared heterogeneity in gene expression data hinders identification of subpopulations present in the phenotype sample and the specific biological factors associated with them. We presented a method, SPSNet, which discovers and analyzes such heterogeneity. As opposed to previous approaches that derived gene-based signatures to identify potential subpopulations within specific diseases, our method is a generic tool which provides subnetwork-based signatures for subpopulations in any phenotype.

While many methods are available for differential expression analysis on homogeneous phenotypes, only a few produce consistent results over independent datasets containing the same phenotypes, and none are designed to deal with potential heterogeneity in the data. PFSNet is one method among the rare exceptions which results in consistent outcomes, but it is designed to analyze only homogeneous phenotypes. We proposed SPSNet, a generalization of PFSNet, which is able to solve an important problem – handling undeclared heterogeneity in gene expression samples by identifying subnetworks associated with hidden subpopulations within phenotypes. The approach also helps recognize and eliminate extrinsic heterogeneity such as batch effects. We demonstrated that SPSNet has high sensitivity, low false-positive rate, high reproducibility, and high biological coherence when analyzing gene expression data with heterogeneity. The method is shown to work on both microarray and RNASeq datasets.

However, there is room for improvement in the design and performance of SPSNet. For example, SPSNet could benefit from a better subnetwork generation scheme. Although the current procedure for generating candidate subnetworks—selecting each gene and its immediate neighbors in a pathway—is a simple way to account for connections between genes in biological pathways, it is relatively naive and results in fragmented components of pathways. Complementing the information in pathways with that extracted from gene expression datasets could possibly lead to generation of subnetworks that are more cohesive and biologically meaningful. Research is also necessary to further improve the sensitivity of SPSNet.

## Additional files

Additional file 1 Human pathways from PathwayAPI [18]. (TXT 2877 kb)

Additional file 2 Rat pathways from KEGG [19]. (TXT 19558 kb)
